# Public engagement in setting healthcare priorities: a ranking exercise in Cyprus

**DOI:** 10.1186/s12962-017-0078-3

**Published:** 2017-08-09

**Authors:** Antonis Farmakas, Mamas Theodorou, Petros Galanis, Georgios Karayiannis, Stefanos Ghobrial, Nikos Polyzos, Evridiki Papastavrou, Eirini Agapidaki, Kyriakos Souliotis

**Affiliations:** 1 Department of Life and Health Sciences, University of Nicosia, Nicosia, Cyprus; 2 Faculty of Economics and Management, Open University of Cyprus, Nicosia, Cyprus; 3 Research Associate Center for Health Services Management and Evaluation, Faculty of Nursing, University of Athens, Athens, Greece; 4 University of Nicosia, Nicosia, Cyprus; 5 School of Medicine, East Anglia University, Norwich, UK; 6 Department of Social Administration and Political Science, Democritus University of Thrace, Komotini, Greece; 7 Department of Nursing, Cyprus University of Technology, Limassol, Cyprus; 8 Centre for Health Services Research, Department of Hygiene, Epidemiology and Medical Statistics, Medical School, University of Athens, 25 Alexandroupoleos st., Athens, Greece; 9 Faculty of Social and Political Sciences, University of Peloponnese, Corinth, Greece

**Keywords:** Public participation, Healthcare priority setting, Resources allocation, Health policy

## Abstract

**Background:**

In countries such as Cyprus the financial crisis and the recession have severely affected the funding and priority setting of the health care system. There is evidence highlighting the importance of population’ preferences in designing priorities for health care settings. Although public preferences have been thorough analysed in many countries, there is a research gap in terms of simultaneously investigating the relative importance and the weight of differing and competing criteria for determining healthcare priority settings. The main objective of the study was tο investigate public preferences for the relative utility and weight of differing and competing criteria for health care priority setting in Cyprus.

**Methods:**

The ‘conjoint analysis’ technique was applied to develop a ranking exercise. The aim of the study was to identify the preferences of the participants for alternative options. Participants were asked to grade in a priority order 16 hypothetical case scenarios of patients with different disease and of diverse socio-economic characteristics awaiting treatment. The sample was purposive and consisted of 100 Cypriots, selected from public locations all over the country.

**Results:**

It was revealed that the *“severity of the disease”* and the “*age of the patient”* were the key prioritization criteria. Participants assigned the smallest relative value to the criterion “*healthy lifestyle”*. More precisely, participants older than 35 years old assigned higher relative importance to “*age”*, while younger participants to the “*severity of the disease”.* The “*healthy lifestyle”* criterion was assigned to the lowest relative importance to by all participants.

**Conclusion:**

In Cyprus, public participation in health care priority setting is almost inexistent. Nonetheless, it seems that the public’s participation in this process could lead to a wider acceptance of the healthcare system especially as a result of the financial crisis and the upcoming reforms implemented such as the establishment of the General System of Health Insurance.

## Background

It is evident that public spending on health is a necessary but not sufficient condition to meet the health care needs and ensure optimal population’ health outcomes [1]. Public spending on health has been increased worldwide, not only due to rising health needs but also because of the medical and pharmaceutical innovation, the research advances and the population shift from treatment to prevention and health promotion [1, 2]. Priority setting may thus constitute an effective approach in order to increase public participation in health policy decision making and shaping sustainable healthcare systems not only in developing countries where health care resources are restricted [2–4] but in all countries, healthcare systems and settings [5].

Countries such as Sweden, Norway, England and Israel have developed guidelines and procedures to engage the public in priority setting [6, 7]. On the other hand, in countries such as Cyprus, Greece [8] and Germany, public participation in health care priority setting is limited [9]. Although that public engagement is strongly related to social justice, efficient and effective health services and better population health outcomes, many countries fail to implement policies and practices for public involvement in healthcare settings prioritization [6].

Research has shown that various factors influence the public involvement in the decision making process for resource allocation and priority setting. Some researchers suggest that age (which holds a prominent position through the years of researching this area of study) [1, 10–15] family size and gender are considered to be the most influential factors in resource allocation and priority setting [14, 16] while others suggest that personal characteristics should not be associated with priority setting [1]. Moreover, *lifestyle* [14], *person’s contribution in causing the disease* [17, 18] *quality of life* and *improvement in health as a result of a treatment* i.e. health gain after treatment [11], *severity of disease* and *cost of treatment* [19, 20] *waiting time* and the *patient’s family responsibilities* appear to have an impact on priority setting [13, 21]. Research has also shown that the public’s differing options may reflect the differences in educational level [10, 14, 18, 22, 23] while other studies do not attribute the differing options to education or personal traits [24].

A number of underlying values and principles are highlighted in the literature regarding the public involvement in healthcare priority setting. Few examples are: (a) necessity [25–27] (b) efficiency [28, 29], (c) of maximization [27], (d) merit [22, 27, 29] and (e) justice [17, 28] or equality or equivalence [27].

The aim of the study was to investigate the public preferences for health care priority setting by using competing criteria (for relative utility and weight) as well as to explore and describe the public views in shaping health care policy. To our knowledge this is the first attempt to investigate public views concerning issues of setting priorities in health care and health policy decision making in Cyprus. It should be noted that the study was carried out in the context of the ongoing financial crisis and the upcoming health reforms. It is anticipated that the findings from our study may contribute to bridge the gap between public participation and health policy decision making and aid the effort to develop sustainable healthcare systems especially across European countries.

## Methods

### Instrument

The current descriptive study was conducted by employing a conjoint-analysis of a ranking exercise to facilitate individuals express their views on specific healthcare aspects towards prioritization [30, 31].

According to the literature, ranking exercise is considered to be a simple, easy to use and accurate method to explore public views and opinions. Specifically, it was used to investigate whether and how the Cypriots consider age, healthy lifestyle, type of disease, severity of disease, health improvement and cost of treatment to be important criteria for resources allocation, as well as to describe their preferences and opinions concerning the importance and usefulness of competing health care priority setting criteria. Moreover, the association between potential influential factors such as gender, age and educational background of the study participants and their views and preferences was also examined.

The conjoint analysis technique was used to elicit participants’ preferences in terms of the alternative options. This technique has been widely used for market research purposes since the 1970s and has gained relative prominence in the field of health care research in recent years [32]. The main advantage of this method is that participants are invited to consider several attributes or criteria concurrently and are thus facilitated to compromise and make trade-offs to reach a decision. In this way, the relative importance and the usefulness of each criterion can be elicited [33]. The conjoint analysis is based on preference judgements and allows for calculating the usefulness of each attribute and its various levels [6, 34].

Τhe rationale for using this method to investigate public preferences for hypothetical patients is documented as follows: (a) it was successfully used in previous studies [6], (b) it would be much easier for the study participants to have realistic scenarios at hand and rank them in priority order, since top priority can only be given to one patient and (c) it is a straight-forward, easy to use method, so participants would be able to decide on the priority rank order [34].

Conjoint analysis for the present study was developed according to the following steps: (a) identifying attributes (variables) and their individual levels, (b) presenting the scenarios, (c) selecting participants and (d) analysing data. The analysis resulted to the study questionnaire. It was constituted from 16 hypothetical cases of patients asking for health care treatment. The criteria and particular traits of patients were the attributes (variables) used for the purposes of this study. Participants were asked to rank order these 16 hypothetical patients based on their priorities for health care treatment.

#### Attributes (variables)

The selection of the criteria used in the present study was based on the following steps:

- A thorough and systematic review of the relevant scientific literature was conducted to facilitate the understanding and formulation of the criteria for healthcare priorities setting. The literature review revealed a number of sixty-three (63) criteria.
- Systematic research team meetings were carried out in order to examine the relevance and importance of the sixty-three criteria identified. Team members discussed each criterion thoroughly aiming to agree on an initial set with a reduced number of criteria. This process resulted to the development of an initial pool of twenty-two criteria (22).
- The initial pool of the twenty-two criteria was reviewed by an experts’ panel in terms of importance and relevance in the context of Cyprus social, cultural, economic and healthcare circumstances. The aim of the experts’ panel was to provide feedback and result in a decreased number (six at a maximum) of the most important and relevant criteria to be used in the study. The experts’ panel was consisted of: (a) members of the public, (b) health care professionals and (c) health policy key-stakeholders. Initially, 32 individuals were recruited to participate voluntarily in the experts’ panel. The selection of potential participants was based on their experience and expertise (e.g. members of the public who were active citizens and engaged to advocacy groups for reducing health inequalities). Finally, thirteen (13) individuals agreed to participate (four members of the public, five health professionals and four health policy key-stakeholders) and joined the experts’ panel. The group of experts was asked to provide feedback about the initial pool of twenty-two criteria and express their opinions on health policy and priority setting in the view of the financial crisis. The criteria were discussed thoroughly in systematic experts’ group meetings in order to examine their relevance and importance, reach consensus and reduce the number of the initial set (of the twenty-three criteria) to six. The experts’ panel confirmed that the final set of cases and attributes were relevant, important, comprehensible and suitable for the Cyprus population. Apart from expressing their views on the topic, participants had also the opportunity to raise questions on the usefulness and the importance of the study in the era of financial crisis.

The following six (6) independent attributes (variables) were included in the conjoint analysis: age, healthy lifestyle, type of disease, severity of disease, improvement in health after treatment and cost of treatment. Each of these attributes had 2–3 levels. It should be noted that literature suggests that the analysis should include up to six (6) attributes (variables) [6, 34] of three (3) levels [35].

### Age

This attribute refers to the patient’s age at onset of illness. For the purposes of the study, age was assigned to three levels: 16, 37 and 68 years of age, to represent minors/adolescents, working people and pensioners respectively.

### Healthy lifestyle

This variable describes the person’s living habits and lifestyle as *healthy* or *unhealthy*, thus it is assigned two levels: “yes” or “no”, respectively. A patient attributed to “healthy lifestyle” is a non-smoker, with moderate alcohol consumption, healthy-eating habits, who is sufficiently exercising. As a patient with an “unhealthy lifestyle” was considered anyone who did not conformed to one or more of these characteristics [6]. Each health behaviour (alcohol consumption, healthy nutrition, etc.) was defined according to the WHO guidelines for healthy lifestyles [36].

### Type of disease

This attribute describes the patient’s disease and has two levels: “chronic” and “acute”. Patients with chronic diseases are those who are diagnosed with a particular disease and receive medication on a regular basis or those who frequently (every 3 months) are in need of medical treatment. As patients with acute diseases were considered those who need medical treatment without suffering from a chronic disease.

### Severity of disease

The “severity of disease” attribute was assigned two levels: “mild” and “severe”. This attribute actually refers to the patient’s health condition before treatment and this is what is classified as “severe” or “mild”.

### Health improvement after treatment

This attribute refers to the anticipated health improvement after treatment. Thus, it refers to the positive health outcomes derived from treatment and the general health gain expected for each patient. The levels assigned to this attribute are: “Low”, “medium” and “high”.

### Cost of treatment

The cost of treatment refers to the out of pocket cost for medical treatment for each patient. Three levels are assigned to this attribute: “low”, “medium” and “high”.

Table 1 summarises the various attributes (variables) and their assigned levels as included in the conjoint analysis ranking exercise.

**Table 1:** Attributes and their assigned levels included in the ranking exercise

Attributes	Description	Levels
Age	Patient’s age at the time s/he got sick	16 years old 37 years old 68 years old
Healthy lifestyle	Characterizes whether the patient is a non-smoker, with mild alcohol consumption, healthy eating habits and sufficient workout, in contrast to a patient who lacks one or more of the abovementioned characteristics	Yes No
Type of disease	The patient’s type of condition	Chronic Acute
Severity of disease	Health status prior to treatment	Mild Severe
Health improvement	Health improvement expected after treatment	Small Mediocre Large
Cost of treatment	Monetary units to be spend for the patient’s treatment	Low Medium High

### The scenarios

Congruent with these, 216 (2^3^ × 3^3^) scenarios were developed to describe hypothetical patients. However, according to the literature plausible scenarios should not exceed 30 [6, 34]. To reduce the number of scenarios, the authors used the SPSS 21.0 procedure [37] to develop a fractional factorial design and reduce the 216 possible scenarios to 16. Winkelhage and Diederich [6] used the same methodology in their research to a sample of German citizens. This model ensures that after the data analysis, the importance of each particular attribute (characteristics) and each level assigned to it will prevail, while all other attributes will remain constant.

### Participants and procedures

A total of 100 participants were selected and asked to rank priority of treatment of 16 hypothetical patients with different characteristics in terms of age, lifestyle, type and severity of disease, expected health gain after treatment and cost of treatment.

The study was conducted between February and September 2015. Study participants were recruited from public locations (e.g. supermarkets and squares) all over Cyprus. A purposive sample of maximum variation was used based on the following criteria: gender (50% men and 50% women), age (50% >35 years old and 50% <35 years old, educational background (the aim was to include people with different educational levels: secondary school, high school and university) and place of residence (urban 73.3%, rural 26.7%).This sampling technique was selected because our aim was to capture a wide range of perspectives relating to the subject, included extreme cases so as to gain deeper understanding on the phenomenon.

Eligible participants were Cypriot citizens, recruited in public areas such as supermarkets, coffee shops, bus stations, markets, squares and streets of the inner city. Potential participants were approached during different hours of the day (morning, afternoon and evenings) so as to ensure maximum variation.

### Data collection

The questionnaire with the 16 case-scenarios was distributed to all participants. The principal investigator made a brief introduction about the study to each individual participated in the study, explained the six attributes and their assigned levels and also provided instructions about the completion of the ranking exercise. The ranking exercise started by explaining participants the six (6) attributes and their assigned levels. Each participant was handed 16 small cards describing each of the 16 scenarios in a table format. Each card basically represented one hypothetical patient and described its main characteristics (Table 2). Individuals were asked to rank order the 16 cards according to priority of treatment. For ease of use, participants were initially asked to sort the 16 cards in three (3) piles: (a) those with the highest priority, (b) those with the lowest priority and (c) those for which they were unsure. Following this, participants had to rank the 16 cards by assigning numbers: starting with number 1 for the card with the highest priority, ending with number 16 for the card with the lowest priority. They were also asked to write down the number on each card after double-checking their decisions. Demographic characteristics (gender, age and educational level) were also obtained.

**Table 2:** Card of a hypothetical patient

Patient 1 rank order	
Age	37 years old
Healthy lifestyle	No
Type of disease	Acute disease
Severity of disease	Mild
Health improvement after treatment	Mediocre improvement
Cost of treatment	Low

### Ethical implications

The study conformed to all the ethical requirements including notification to the authority responsible for the protection of the personal data, the authors secured a permission from the Office of the Commissioner for personal data protection of the Republic of Cyprus to set up and maintain records for this study. Written informed consent was obtained from all participants. Anonymity, confidentiality and voluntary participation were assured and the data were kept in a safe place and used only for the purpose of the study.

### Statistical analysis

Data analysis was based on the conjoint analysis technique. The attributes that participants took into consideration and assigned relative importance were the following: severity of disease, age, type of disease, health improvement after treatment, cost of treatment and healthy lifestyle. These six (6) attributes were used as discrete variables. Sixteen (16) preference cards were created based on the six (6) abovementioned attributes and participants ranked order those cards in terms of utility (starting with the most preferred hypothetical patient to the least important hypothetical patient). Using conjoint analysis the relative utility of each of the six (6) attributes was elicited along with their relevant values with highest values indicating high utility and high relative value. Utility (Χ) for each patient profile is derived from the following equation [6]:

$${\rm X} \, = {\text{ the model's constant }} + \, {\rm X}_{\text{age}} + \, {\rm X}_{\text{healty lifestyle}} + \, {\rm X}_{\text{type of disease}} + \, {\rm X}_{\text{severity of disease}} + \, {\rm X}_{\text{health improvement}} + \, {\rm X}_{\text{cost of treatment}} .$$

To test the correlations between various utilities and the gender, age and educational level of the study participants the authors used the *t* test. Also, we performed analysis of variance and *t* test in order to assess differences on relative importance ascribed by the participants to the six characteristics of the study, based on their gender, age and educational level. The two-sided significance level was set equal to 0.05. Data analysis was conducted using IBM SPSS 21.0 (Statistical Package for Social Sciences).

## Results

### Demographic characteristics of the sample

The study sample consisted of 50 women (50%) and 50 men (50%). 23% (n = 23) were secondary school graduates, 38% (n = 38) were high school graduates and the remaining 39% (n = 39) of the study population were University graduates. The mean, standard deviation and median of the participant’s age was 38.9, 14.3 and 36 years of age, respectively. The minimum and the maximum values in terms of age were 18 and 74, respectively.

The model’s Pearson correlation coefficient was 0.993 (p < 0.001), which indicates fairly good internal validity for the study.

Utilities and relative values (in terms of importance) ascribed by the study participants to the six (6) attributes.

Table 3 summarises the utilities and relative importance ascribed by participants to the six (6) attributes of this particular study. It becomes evident that younger patients, unhealthy lifestyle, acute and severe disease, large improvement in health and low cost of treatment are assigned with highest levels of utility.

**Table 3:** Utilities and relative importance ascribed by the participants to the six (6) attributes of the study

Attribute	Categories	Utility (standard error)	Relative importance
Age	16 years old	1.427 (0.142)	25.5
	37 years old	−0.018 (0.167)	
	68 years old	−1.408 (0.167)	
Healthy lifestyle	Yes	−0.249 (0.107)	7.9
	No	0.249 (0.107)	
Type of disease	Chronic	−0.388 (0.107)	15.2
	Acute	0.388 (0.107)	
Severity of disease	Mild	−2.046 (0.107)	27.4
	Severe	2.046 (0.107)	
Health improvement	Mediocre	−0.198 (0.107)	12.1
	Large	0.198 (0.107)	
Cost of treatment	Low	0.223 (0.142)	11.9
	Medium	−0.127 (0.167)	
	High	−0.097 (0.167)	
Constant		8.088 (0.118)	

Ranking attributes/characteristics according to their relative importance (value), starting with the characteristic with the highest relative importance come down to the following:

- Severity of disease
- Age
- Type of disease
- Health improvement after treatment
- Cost of treatment
- Healthy lifestyle

The maximum utility (thus, importance) was ascribed to the patient demonstrating the following characteristics:

- 16 years of age
- Unhealthy lifestyle
- Acute disease
- Severe disease
- Large health improvement after treatment
- Low cost of treatment

The total maximum utility for the above-mentioned patient is calculated as follows:

$$maximum \;utitlity = 8.088 + 1.427 + 0.249 + 0.388 + 2.046 + 0.198 + 0.223 = 12.619$$

The lowest level of utility was ascribed to the patient demonstrating the following characteristics:

- 68 years of age
- Healthy lifestyle
- Chronic disease
- Light disease
- Mediocre health improvement after treatment
- High cost of treatment

The total lease utility for the above-mentioned patient is calculated as follows:

$$least\;utility = 8.088 - 1.408 - 0.249 - 0.388 - 2.046 - 0.198 - 0.127 = 3.672$$

Table 4 summarises the relative importance values ascribed by participants to the six (6) attributes of this particular study based on their gender, age and educational level.

**Table 4:** Relative importance ascribed by the participants to the six (6) characteristics of the study, based on their gender, age and educational level

Characteristic	Relative importance											
	Age	p value	Healthy lifestyle	p value	Type of disease	p value	Severity of disease	p value	Health improvement	p value	Cost of treatment	p value
Gender		*<0.001* ^a^		*0.04* ^a^		*<0.001* ^a^		*0.002* ^a^		*<0.001* ^a^		*<0.001* ^a^
Women	28.3		8.2		12.5		27.8		13.0		10.2	
Men	22.7		7.7		17.9		27.0		11.2		13.5	
Age (years)		<*0.001* ^*a*^		0.67^a^		<*0.001* ^a^		<*0.001* ^a^		0.11^a^		<*0.001* ^a^
≤35	22.7		8.0		13.7		29.6		12.3		13.7	
>35	28.2		7.9		16.7		25.2		11.9		10.1	
Educational level		<*0.001* ^b,c^		<*0.001* ^b,d^		<*0.001* ^b,e^		**<** *0.001* ^b,d^		<*0.001* ^b,f^		<*0.001* ^b,f^
Secondary school (‘gymnasium’) graduates	32.0		7.4		11.0		25.4		11.4		12.8	
High school graduates	23.6		7.0		17.1		28.2		12.1		12.0	
University graduates	23.4		9.1		15.9		27.9		12.5		11.2	

^a^ *t* test

^b^Analysis of variance

^c^Statistical significant difference between secondary school graduates and high school graduates (p = 0.049)

^d^Statistical significant difference between secondary school graduates and high school graduates (p < 0.001) and secondary school graduates and university graduates (p < 0.001)

^e^Statistical significant difference among the three groups (p < 0.001 in all cases)

^f^Statistical significant difference between secondary school graduates and high school graduates (p < 0.001)

Utilities ascribed by the study participants to the six (6) attributes based on the participants’ gender, age and educational level.

The results indicate that women ascribe the largest relative importance to age, while men opted for the severity of the disease. Both women and men ascribed the lowest level of relative importance to healthy lifestyle. Women gave priority to younger patients, in contrast to men. Men gave priority to a patient with large health improvement after treatment, in contrast to women. All differences according to gender were statistically significant.

Participants with more than 35 years of age ascribed the highest level of relative importance to age (p < 0.001), while participants less than 35 years of age opted for the severity of the disease (p < 0.001). Both groups, however, ascribed the least relative value to healthy lifestyle (p = 0.67). Moreover, participants older than 35 gave priority to a 16 years old patient, in contrast to those participants with less than 35 years of age (p < 0.001).

Participants with the lowest educational level assigned high importance to younger patients and to the “health improvement after treatment” attribute, in comparison to the participants with the higher educational level who gave priority to a patient with acute disease (p < 0.001 in all cases). Secondary school graduates ascribed the highest level of importance to age, while high school and University graduates to the severity of the disease. The lowest value in terms of importance was assigned to “healthy lifestyle”, irrespective of the participants’ educational level.

## Discussion

The purpose of the present study was to investigate preferences and the relative importance of possible criteria that could be taken into consideration for health care priority setting in Cyprus. This is the first attempt on the topic in Cyprus and thus there are no similar data available to benchmark against and compare the results.

Health care priority setting in Cyprus does not take into consideration public preferences, as is the case in other countries and this seems to be the case in Germany as well, based on the findings of a similar study [6].

Results from the present study revealed that when the citizens asked to set priorities in health care, they report the “severity of disease”, “age”, “type of disease”, “health improvement after treatment”, “cost of treatment” and “healthy lifestyle” are all possible criteria that should be considered. This is congruent to similar findings of other studies [6].

The utilities and relative importance values ascribed by the 100 respondents to the six (6) attributes of the study indicate that high priority and thus high utility is associated with patients that demonstrate young age, unhealthy lifestyle, acute and severe diseases, large expected health improvement after treatment and low cost of treatment. Thus, ranking the patients’ characteristics by their relative importance value, starting with the one with the highest importance, elicits the profile of the hypothetical patients with the highest and the lowest priority.

The study findings indicate that participants gave the *highest* priority to a patient of 16 years of age, with unhealthy lifestyle, an acute disease that is also severe, who is expected to have large health improvement after treatment and, the cost of his/hers treatment is low. On the contrary, *lowest priority* is given to a 68 years old patient, with healthy lifestyle and a chronic disease of mild form expected to have mediocre improvement to his/her health condition after treatment and whose cost of treatment is high.

Moreover, it was identified that the “severity of disease”, “age” and “type of disease” are the three (3) attributes that have the highest importance values. The first two factors although in different order were also found in the study of Winkelhage and Diederich [6] in Germany to influence public preferences in healthcare priority setting. In their study, the most important was age and then severity of the disease. The finding that the “severity of disease” is an important parameter for priority setting and resource allocation in health care is in line with the findings of other studies [18, 19, 23, 28]. Our findings are in line with previous studies indicated that the severity of the disease was either an important parameter for resource allocation in health [1, 18, 19, 23] or was strongly supported by the public as an important health care priority setting criterion [6, 15, 23, 28, 38]. Moreover, the study findings indicate that priority should be given to patients with a severe disease or urgent conditions. The opinions of people participating in the study chose criteria that are mostly concerned with issues related to the value of life, social justice and equality [1].

Having as a starting point the fact that participants gave priority to people in greater need for health care treatment can only lead to the assumption that the ground of this particular choice is based on the principle of necessity. These preferences are rather justified and expected since they raise issues that relate to the ultimate value, that of life itself. In life or death situations, the public is not expected to make choices in favor of the sustainability of the health care system or in favor of value maximization, but are rather expected to make choices that can be explained in terms of the principle of necessity. The principle of necessity actually entails that health care services should be offered to the public according to the actual “need” [28, 29, 39]. This approach defines “need” as the severity of the disease [40] and supports that what should also be taken into consideration is the “urgency of the situation”. Therefore, those who are suffering from severe diseases should be priority ranked for health care treatments. This principle seems to be highly supported in Scandinavian countries. The severity of the situation, in the sense that the person who is in greater need should receive health care treatment first, is actually the first priority criterion in the Swedish and Norwegian laws [7, 41–45].

### Age

In the present study “age” was identified the second most significant attribute in terms of utility and importance. Results indicated that older participants gave priority to younger patients. This decision might be based on internal motives and interests [46]. Our findings are in line with the those of other similar studies [6, 47] which used the same conjoint analysis methodology and support consistently that young people should have priority over others for health care treatment [6, 11, 23, 48–54]. On the other hand, other researchers suggest that age should not be a priority setting criterion or yield small support in its favor [1, 10, 11, 14, 18, 19, 21, 38, 55–60]. This implies that these findings may reflect the fact that the same methodology was used in all these studies. This premise, that the order of the questions and the nationality of the participants seem to affect the research results, is also evident in other studies [5, 14, 15, 20, 51, 61–63].

The attribute of age in our study was presented with three concrete levels (16, 37 and 68 years), while the others were described rather abstractedly with levels such as “mild” or “severe”. One may suggest that this might have influenced the results. However, it seems that the variables ordering did not impacted the results, since the rank order in terms of utility differs from the ones presented in the 16 scenario cards. The fact that “age” was assigned with the second highest utility level needs further investigation, especially because is in conflict with the European directive for equality in health care that suggests that chronological age is less important than biological age [55].

Evidence suggests [6], elements such as younger patients, patients with larger expected health improvement after treatment and low cost treatments, are associated with the “principle of efficiency”, while supporting healthy lifestyle in terms of health care priority is associated with the “principle of merit”. The hypothesis of their research was that participants with higher educational level would be supportive of the principles of efficiency and merit, in contrast to participants with lower educational background. However, this hypothesis was confirmed neither from their study nor from the present study. Instead, participants with lower education support importance values that are associated with the efficiency principle. It should also be noted that other researchers reached the conclusion that accepting “age” as a criterion for health care resource allocation is not associated with the respondents’ educational level [14].

### Healthy lifestyle

The “healthy lifestyle” attribute in the present study was not supported by participants with higher education, in contrast to other studies [6, 22]. According to Winkelhage and Diederich [6] this criterion corresponds to the principle of merit. Participants assigned the lowest relative importance value to “healthy lifestyle” while at the same time ascribed utility to the “unhealthy lifestyle” variable.

According to Myllykangas et al. [64] if lower priority is given to people with unhealthy lifestyle this will result to increased social health inequities since these population groups are in greater need for medical treatment. The literature suggests that health care resource allocation based on lifestyle is much debated and rather problematic. The adoption of a healthy lifestyle is also influenced by education [65] which is associated with the socioeconomic status [66] which in turns affects the person’s health status consisting a vicious cycle. It is assumed that people of high educational level are most likely to support the *principle of necessity* compared to people of low, since this principle actually underlines that individual contributions should be rewarded [6].

Cypriots in the present study gave priority to patients with unhealthy lifestyle as opposed to those with healthy lifestyle, irrespective of age, gender and educational level. A possible explanation is that respondents considered people with an unhealthy lifestyle to be in greater need for medical treatment. Another plausible interpretation is that unhealthy lifestyle is highly associated with risk factors such as smoking, obesity and physical inactivity. Considering that Cyprus ranks high in tobacco consumption between EU countries as well as in physical inactivity and obesity, one may assume that a large proportion of the participants had one or more risk factors and this might have affected their decision-making. Irrespective of these assumptions, this finding may suggest that people of low socio-economic status and poor health should have priority due to social inequalities in health. It seems that participants’ responses were based on the principle of necessity or at least we can pertain that these findings can be partially explained on the grounds of this principle.

### Policy implications

Findings from the present study may assist health policy makers in their effort to strengthen the health system and set priorities. Of course, the list of criteria (“age”, “type of disease”, “health improvement after treatment”, “cost of treatment” and “healthy lifestyle”) is not exhaustive but indicative. The integration of public preferences into health policy decision making processes requires a systematic, reliable and effective strategy of public participation. The contribution of citizens to health policy decision making should be official and continuing: from planning to evaluation of the health services and programs. It should be noted that public preferences is rather complementary than conflicted to evidence based data, since they are based on them and do not apply to every aspect and every area or activity of the healthcare system. For instance, the criterion of “type of disease” may be useful to set priorities in universal screening for non-communicable diseases (e.g. cancer and cardiovascular disease instead of multiple sclerosis) for the general population but perhaps it is not suitable in setting priorities in innovative pharmaceutical therapies and health technology assessment procedures. Thus, specific criteria and strategies should be implemented in the different areas of health policy decision making. Of course, the strategy of public participation is a necessary but not sufficient condition to translate evidence into policies.

There are different levels of health policy decision making (local, national, European) and healthcare priority setting requiring different channels of participation. At a local level, the integration of public opinions is more feasible, due to community based non-governmental organizations which advocate for citizens’ health needs and may be beneficial in consulting local committees and authorities in healthcare priorities setting. At a national level, more solid and official methods (e.g. legislation establishing patient participation) are needed in order to incorporate public views and preferences in health policy decision making. To achieve this goal, academic research would be more community oriented without losing its credibility. An important prerequisite is to increase the productive interaction between researchers, policy makers, advocates and general public. Community based research may facilitate this process. Moreover, it is imperative for researchers to present evidence in a way that can be exploited by policy makers and provide continuing support to every step of the integration procedure. On the other hand, policy makers should develop official mechanisms and strategies so as to ensure that the role of other key stakeholders (e.g. general public, researchers) is not just consultative but impacts the development, implementation and evaluation of health policies, programs and services.

## Conclusions

The incorporation of public opinions in health priority setting and health policy decision making is a challenging process. There is a growing body of evidence suggesting that public participation in health policy decision making is associated to health systems performance and sustainability, but the strategy to achieve it remains an under-investigate issue [6]. Results from the present study revealed that the “severity of disease”, “age”, “type of disease”, “health improvement after treatment”, “cost of treatment” and “healthy lifestyle” should be considered as criteria in healthcare priorities setting. Moreover, it was identified that “severity of disease”, “age” and “type of disease” were the attributes with the highest importance. This may be attributed to the fact that the study was carried out during the financial crisis in Cyprus which implies that health care needs limited resources. A critical point for the integration of public preferences in health policy decision making is whether they lead to increase or reduce of inequalities in health [2]. Since vulnerable groups are affected disproportionally by health inequalities they should have a say in healthcare priority setting. Thus, it should be clearly defined what we mean by the term “public participation”. Are vulnerable and disadvantaged groups included or just the general population? On the other hand, we should not be focused only on vulnerable groups, because the healthcare system should be accessible and effective for all. This implies that different sources of information and different strategies of data collection should be implemented in order to ensure that all population’ groups are included and the data collected are representative and reliable.

## Study limitations

The fact that interactions between the various attributes in pairs or in triplets were not analyzed is a methodological limitation of the present study, especially considering that Rodriguez and Pinto revealed that age interacts with health gain associated with medical treatment [52]. Nonetheless, the design of the present study did not allow for the analysis of these interactions because the patient scenarios would increase and become unmanageable for the participants.

The small and purposeful study sample is implies further limitations but the purpose of the study was not the generalizability of the results but the provision of critical information.

Another study limitation is that participants had rather limited information at their disposal. One can assume that should participants had the opportunity to secure adequate and substantial information and to discuss all relevant parameters of the study they might have reached different decisions. To downsize this limitation, the authors provided all participants with the same information and answered all their questions.
